# Oncology Clinicians’ Attitudes on Hormonal Therapy After Chemoradiotherapy for Cervical Cancer

**DOI:** 10.1001/jamanetworkopen.2026.6862

**Published:** 2026-04-14

**Authors:** Morgan S. Levy, Marilyn Huang, Charles S. Dietrich, Denise Fabian

**Affiliations:** 1 Department of Radiation Oncology, University of Kentucky Markey Cancer Center, Lexington; 2 Department of Gynecologic Oncology, University of Virginia, Charlottesville; 3 Department of Gynecologic Oncology, University of Kentucky Markey Cancer Center, Lexington

## Abstract

This survey study assesses gynecologic oncology and radiation oncology clinician attitudes towards, patterns of, and barriers to prescribing hormonal therapy after chemoradiotherapy for cervical cancer.

## Introduction

Premenopausal patients with locally advanced cervical cancer (LACC) treated with chemoradiotherapy often experience iatrogenic menopause due to ovarian ablation from radiotherapy.^1^ Hormonal therapy (HT) safely and effectively treats menopausal symptoms after pelvic radiotherapy.^2,3^ Despite clinical practice guidelines recommending HT, it remains underused.^1,4^ This survey study assesses gynecologic oncology (GYO) and radiation oncology (RO) clinician attitudes, HT prescribing patterns, and barriers to prescribing HT after chemoradiotherapy for cervical cancer.

## Methods

For this survey study, we administered a questionnaire about HT for patients with LACC after chemoradiotherapy, following American Association for Public Opinion Research (AAPOR) reporting guidelines, to GYO and RO physicians and advanced practice professionals from April 23 to June 9, 2025. Participants were recruited through the listservs of Society of Gynecologic Oncology (SGO), the American Brachytherapy Society (ABS), and experts in cervical cancer brachytherapy. The survey collected demographic characteristics, cervical cancer practice patterns, and instances of prescribing HT. Race and ethnicity were self-reported. The study was approved by the University of Kentucky institutional review board, and participants provided written informed consent. Statistics were calculated using IBM SPSS, version 30 (IBM Corp), using the χ^2^ and Fisher exact tests, with analyses conducted by specialty to assess practice differences. All *P* values were from 2-sided tests and results were deemed statistically significant at *P* < .05.

## Results

Of 209 participants who started the survey (9.4% of 1490 eligible SGO and 0.3% of 10 000 ABS), 186 (89.0%) completed it and 178 (95.7%) were eligible (Table 1). Participants included members of SGO (75.3% [n = 140]), ABS (16.7% [n = 31]), and gynecologic RO experts (3.8% [n = 7]). Most were attending physicians (GYO, 77.9% [106 of 136]; RO, 81.0% [34 of 42]) and women (GYO, 76.5% [104 of 136]; RO, 59.5% [25 of 42]) and see 1 to 5 patients with cervical cancer per month (GYO, 58.8% [80 of 136]; RO, 57.1% [24 of 42]). Most of the participants always discuss iatrogenic menopause with patients before chemoradiotherapy (GYO, 62.5% [85 of 136]; RO, 78.6% [33 of 42]).

**Table 1.** Participant Demographic Characteristics and Practice Patterns by Specialty

Characteristic	Oncology, No. (%)		χ^2^ Value (*df*)	*P* value
	Gynecologic (n = 136 [76.4%])	Radiation (n = 42 [23.6%])		
Title				
Attending physician	106 (77.9)	34 (81.0)	29.951 (3)	<.001
Fellow physician	12 (8.8)	0		
Resident physician	0	7 (16.7)		
Advanced practice professional	18 (13.2)	1 (2.4)		
Gender				
Woman	104 (76.5)	25 (59.5)	6.012 (2)	.049
Man	30 (22.1)	17 (40.5)		
Prefer not to answer	2 (1.5)	0		
Race and ethnicity^a^				
American Indian or Alaska Native^b^	1 (0.7)	1 (2.4)	NA	.38
Asian^b^	12 (8.8)	14 (33.3)	NA	<.001
Black or African American^b^	9 (6.6)	1 (2.4)	NA	.46
Hispanic, Latino, or Spanish^b^	9 (6.6)	1 (2.4)	NA	.46
White	102 (75.0)	24 (57.1)	4.948 (1)	.03
Other^b^^,^^c^	2 (1.5)	1 (2.4)	NA	.56
Prefer not to answer^b^	5 (3.7)	3 (7.1)	NA	.40
Years in practice since completion of training				
Currently in training	12 (8.8)	6 (14.3)	1.846 (4)	.76
≤5	37 (27.2)	8 (19.0)		
6-10	29 (21.3)	9 (21.4)		
11-20	30 (22.1)	10 (23.8)		
≥21	28 (20.6)	9 (21.4)		
On average, how many patients with cervical cancer do you see each month?				
0	1 (0.7)	0	0.756 (3)	.86
1-5	80 (58.8)	24 (57.1)		
6-10	35 (25.7)	10 (23.8)		
>10	20 (14.7)	8 (19.0)		
How often do you discuss iatrogenic menopause with patients with cervical cancer who will undergo chemoradiotherapy?				
Always	85 (62.5)	33 (78.6)	4.149 (4)	.39
Most of the time	38 (27.9)	6 (14.3)		
About half the time	5 (3.7)	1 (2.4)		
Sometimes	7 (5.1)	2 (4.8)		
Never	0	0		
Upon patient request	1 (0.7)	0		
In your experience, what percentage of premenopausal patients treated with chemoradiotherapy experience menopausal symptoms after treatment?				
All	26 (19.1)	8 (19.0)	1.154 (4)	.87
Most	81 (59.6)	24 (57.1)		
Some	27 (19.9)	9 (21.4)		
A few	1 (0.7)	0		
None	1 (0.7)	1 (2.4)		

Abbreviation: NA, not applicable.

^a^ Participants were able to choose more than 1 response.

^b^ The Fisher exact test was used for analysis because the cell size was less than 15.

^c^ Participants who selected “other” had an option to write in free-text responses. Two participants wrote in a response (“mixed” and “Middle Eastern”), and 1 participant did not enter any text. An option for Native Hawaiian or Pacific Islander was also included but was not selected by any participants.

Most clinicians reported that patients sometimes ask about HT (GYO, 54.4% [74 of 136]; RO, 73.8% [31 of 42]) (Table 2). Almost all GYO clinicians (99.3% [135 of 136]) would prescribe HT after chemoradiotherapy compared with 73.8% of RO clinicians (31 of 42). Among clinicians who would consider HT, most recommended combined estrogen and progesterone in an oral or topical form (GYO, 91.2% [124 of 136]; RO, 70.0% [28 of 40]). There were no significant differences in willingness to consider prescribing HT by race and ethnicity, gender, practice region, mean number of cervical cancer patients treated per month, or years in practice.

**Table 2.** Hormonal Therapy Prescribing Patterns and Barriers

Question	Oncology, No./total No. (%)		χ^2^ Value (*df*)	*P* value
	Gynecologic (n = 136 [76.4%])	Radiation (n = 42 [23.6%])		
In your practice, how often do patients with cervical cancer treated with chemoradiotherapy ask about hormonal therapy at the completion of treatment?				
Always	1/136 (0.7)	0	8.118 (4)	.09
Most of the time	21/136 (15.4)	2/42 (4.8)		
About half the time	29/136 (21.3)	4/42 (9.5)		
Sometimes	74/136 (54.4)	31/42 (73.8)		
Never	11/136 (8.1)	5/42 (11.9)		
Would you consider treating a patient with cervical cancer treated with chemoradiotherapy with hormonal therapy?				
Yes	135/136 (99.3)	31/42 (73.8)	33.165 (2)	<.001
Maybe	1/136 (0.7)	9/42 (21.4)		
No	0	2/42 (4.8)		
What types of hormonal therapy would you consider?^a^^,^^b^				
Estrogen only: oral or topical	37/136 (27.2)	10/40 (25.0)	1.470 (1)	.23
Estrogen and progesterone combined: oral or topical	124/136 (91.2)	28/40 (70.0)	14.822 (1)	<.001
Vaginal estrogen only	50/136 (36.8)	25/40 (62.5)	0.767 (1)	.38
Other	5/136 (3.7)	2/40 (5.0)	0.108 (1)	.74
How likely are you to consider that a patient was premenopausal at time of treatment for hormonal therapy prescribing?				
Extremely unlikely	1/136 (0.7)	0	24.880 (4)	<.001
Somewhat unlikely	2/136 (1.5)	0		
Neither likely nor unlikely	0	2/40 (5.0)		
Somewhat likely	25/136 (18.4)	20/40 (50.0)		
Extremely likely	108/136 (79.4)	18/40 (45.0)		
What specialties do you feel are responsible for prescribing hormonal therapy in patients with cervical cancer who have been treated with chemoradiotherapy?^a^^,^^b^				
Gynecologic oncology	132/136 (97.1)	36/40 (90.0)	3.550 (1)	.06
Radiation oncology	20/136 (14.7)	18/40 (45.0)	16.756 (1)	<.001
Primary care	45/136 (33.1)	11/40 (27.5)	0.445 (1)	.51
Menopause specialist	95/136 (69.9)	22/40 (55.0)	3.060 (1)	.08
Other	9/136 (6.6)	1/40 (2.5)	0.978 (1)	.32
If you are interested in prescribing hormonal therapy to this population but do not prescribe it as often as you would like to, why is that the case?^b^				
Practice too busy	3/136 (2.2)	2/40 (5.0)	9.078 (4)	.06
Capacity to manage long term	29/136 (21.3)	17/40 (42.5)		
Lack of society guidelines	17/136 (12.5)	5/40 (12.5)		
Concern for recurrence of cancer	6/136 (4.4)	1/40 (2.5)		
Prefer to describe	6/136 (4.4)	11/40 (27.5)		

^a^ Participants were able to choose more than 1 response.

^b^ Participants who responded “yes” or “maybe” to being willing to prescribe hormonal therapy answered these items.

Clinicians who did not prescribe HT as often as desired cited barriers, such as capacity for long-term management (GYO, 21.3% [29 of 136]; RO, 42.5% [17 of 40]) and lack of society guidelines (GYO, 12.5% [17 of 136]; RO, 12.5% [5 of 40]) (Table 2). Most clinicians considered whether a patient was premenopausal before chemoradiotherapy in prescribing HT (GYO, 97.1% [132 of 136]; RO, 95.0% [38 of 40]).

## Discussion

In this survey study, most clinicians reported that they would consider prescribing HT to patients with LACC (GYO, 99.3%; RO, 78.3%) after chemoradiotherapy, but implementation barriers remain. Clinicians may be unaware of existing guidelines, as indicated by a survey showing that only 59% were aware of the SGO guidelines, with 46% changing their prescribing practice after the publication.^5^ In a different survey on gynecologic cancer survivors, the most commonly reported barrier to prescribing HT was insecurity in prescription.^6^ Future work will include efforts to increase awareness of existing practice guidelines, quality improvement to assess ways to enhance guidelines to increase clinician comfort with prescribing, and further qualitative work to understand prescribing barriers faced by clinicians.

Limitations of this study include self-selection bias, recall bias, sampling bias, low response rate, and small RO clinician sample. No item directly asked clinicians if they prescribe and manage HT in their own practice. Although evidence suggests that HT is safe for cervical cancer survivors, a gap exists between knowledge and practice, leading to suboptimal survivorship care.

## Notes

Supplement.
Data Sharing Statement

## References

[1] Suzuki Y, Huang Y, Ferris J, Kulkarni A, Hershman D, Wright JD. Prescription of hormone replacement therapy among cervical cancer patients with treatment-induced premature menopause. Int J Gynecol Cancer. 2023;33(1):26-34. doi:10.1136/ijgc-2022-003861 36543392

[2] Kirchheiner K, Smet S, Jürgenliemk-Schulz IM, ; EMBRACE Collaborative Group. Impact of vaginal symptoms and hormonal replacement therapy on sexual outcomes after definitive chemoradiotherapy in patients with locally advanced cervical cancer: results from the EMBRACE-I study. Int J Radiat Oncol Biol Phys. 2022;112(2):400-413. doi:10.1016/j.ijrobp.2021.08.036 34478833

[3] Ploch E. Hormonal replacement therapy in patients after cervical cancer treatment. Gynecol Oncol. 1987;26(2):169-177. doi:10.1016/0090-8258(87)90270-8 2433195

[4] Sinno AK, Pinkerton J, Febbraro T, . Hormone therapy (HT) in women with gynecologic cancers and in women at high risk for developing a gynecologic cancer: a Society of Gynecologic Oncology (SGO) clinical practice statement. Gynecol Oncol. 2020;157(2):303-306. doi:10.1016/j.ygyno.2020.01.035 32067815

[5] McDowell JL, Strawderman M, Betstadt SJ, Moore RG. Estrogen therapy in patients with gynecologic cancer: a survey of gynecologists and oncologists in the United States. Menopause. 2026;33(2):161-166. doi:10.1097/GME.0000000000002643 40924876

[6] Teves M, Palma F, Fatela A, Correia L. Barriers to prescription of hormonal contraception and hormone replacement therapy in gynecological cancer survivors: results of a survey and literature review. J Gynecol Obstet Hum Reprod. 2025;54(3):102902. doi:10.1016/j.jogoh.2024.102902 39730066

