# Supplementary material for: Oncology Clinicians’ Attitudes on Hormonal Therapy After Chemoradiotherapy for Cervical Cancer
Source: JAMA Netw Open. 2026 Apr 14;9(4):e266862. doi: 10.1001/jamanetworkopen.2026.6862 (PMC13080543; doi:10.1001/jamanetworkopen.2026.6862)
Supplement: Supplement. — Data Sharing Statement [file jamanetwopen-e266862-s001.pdf]

Data Sharing Statement

Levy. Oncology Clinicians' Attitudes on Hormonal Therapy After Chemoradiotherapy for Cervical Cancer. *JAMA Netw Open*. Published April 14, 2026.
doi:10.1001/jamanetworkopen.2026.6862

Data
Data available: No

Additional Information
Explanation for why data not available: Data can be made available upon request.
